# Assessing Implementation of Maternal and Perinatal Death Surveillance and Response in Rwanda

**DOI:** 10.3390/ijerph17124376

**Published:** 2020-06-18

**Authors:** Edwin Tayebwa, Felix Sayinzoga, Jacqueline Umunyana, Kusum Thapa, Efugbaike Ajayi, Young-Mi Kim, Jeroen van Dillen, Jelle Stekelenburg

**Affiliations:** 1 IntraHealth International, P.O. Box 6639 Kigali, Rwanda; jumunyana@intrahealth.org; 2 University Medical Centre Groningen, Department of Health Sciences, Global Health, University of Groningen, 9700 RB Groningen, The Netherlands; j.stekelenburg@umcg.nl; 3 Rwanda Biomedical Center, KG 203 St, Kigali, Rwanda; felix.sayinzoga@rbc.gov.rw; 4 Jhpiego, An Affiliate of Johns Hopkins University, Baltimore, MD 21231, USA; kusum.thapa@jhpiego.org (K.T.); Gbaike.Ajayi@icf.com (E.A.); young-mi.kim@jhpiego.org (Y.-M.K.); 5 Maternal and Child Survival Program, Washington, DC 20036, USA; 6 Amalia Children’s Hospital, Radboudumc Nijmegen, 6500 HB Nijmegen, The Netherlands; jeroen.vandillen1@radboudumc.nl; 7 Department of Obstetrics and Gynaecology, Leeuwarden Medical Centre, 8934 AD Leeuwarden, The Netherlands

**Keywords:** Audit, maternal, perinatal, death, surveillance, response, implementation, Rwanda

## Abstract

Maternal deaths remain a major public health concern in low- and middle-income countries. Implementation of maternal and perinatal deaths surveillance and response (MPDSR) is vital to reduce preventable deaths. The study aimed to assess implementation of MPDSR in Rwanda. We applied mixed methods following the six-step audit cycle for MPDSR to determine the level of implementation at 10 hospitals and three health centers. Results showed various stages of implementation of MPDSR across facilities. Maternal death audits were conducted regularly, and facilities had action plans to address modifiable factors. However, perinatal death audits were not formally done. Implementation was challenged by lack of enough motivated staff, heavy workload, lack of community engagement, no linkages with existing quality improvement efforts, no guidelines for review of stillbirths, incomplete medical records, poor classification of cause of death, and no sharing of feedback among others. Implementation of MPDSR varied from facility to facility indicating varying capacity gaps. There is need to integrate perinatal death audits with maternal death audits and ensure the process is part of other quality improvement initiatives at the facility level. More efforts are needed to support health facilities to improve implementation of MPDSR and contribute to achieving sustainable development goal (SDG) 3.

## 1. Introduction

Maternal deaths remain a major public health concern especially in low- and middle-income countries. Approximately 295,000 women died during pregnancy and childbirth in 2017 globally, and most (66%) were from sub-Saharan Africa alone [1]. Overall, maternal mortality ratios in sub-Saharan Africa (SSA) vary between and within countries from 250 to 700 per 100,000 live births [2,3,4,5,6]. In SSA, maternal death is mainly caused by hemorrhage, hypertensive disorder, abortion, and obstructed labor [3,6,7], which is attributed to many factors like socio-demographic, inadequately skilled attendants, poorly functioning health systems, and reduced government health expenditure per capita [3,6,8]. 

Regarding perinatal mortality in SSA, about 70 and 56 deaths per 1000 live births occurred at home and in health facilities, respectively [9]. Early neonatal mortality varied between 27 to 32 per 1000 live births [3,8], while the majority of under-five mortality occurred during infancy [8,9]. 

Rwanda achieved the millennium development goal (MDG) 4-related to reduction of child mortality and 5-related to reduction of maternal mortality in 2015 [3,7], but there is a need to sustain these gains through implementation of evidence-based interventions to further reduce preventable deaths. The maternal mortality ratio decreased from 476 in 2010 to 210 per 100,000 live births in 2015 and the infant mortality rate went down from 50 in 2010 to 32 per 1000 live births in 2015. Also, neonatal mortality went down from 27 in 2010 to 20 per 1000 live births in 2015 [10,11]. In 2015, 1100 women died during pregnancy or from childbirth related causes that had occurred at district hospitals (72%), referral hospitals (21%), and health centers (7%) [12]. Postpartum hemorrhage was the leading direct cause (23%), followed by obstructed labor (12%). Indirect causes accounted for 26% of maternal deaths, with malaria as the leading indirect cause (8%) [12]. The remaining 4% were due to unknown causes. Perinatal mortality was due to intrapartum-related complications (33%), preterm birth complications (28%), and neonatal infections including sepsis and pneumonia (25%) [13]. The time of labor and day of birth is when 40% of all stillbirths and neonatal deaths occur; an estimated 73% of all neonatal deaths occur in the first week of life [13]. Another study conducted in Rwanda showed that neonatal pneumonia, birth asphyxia, prematurity, meningitis, encephalitis, as well as neonatal sepsis were the major causes of perinatal mortality [14]. 

A vital component of any elimination strategy is a surveillance system that can track the number of deaths and provide information about cause of death, underlying contributing factors, and actions to address the contributing factors to prevent future preventable deaths. One of the key actions recommended by both World Health Organization (WHO) and United Nations Children’s Fund (UNICEF) is the institutionalization of Maternal and Perinatal Death Surveillance and Response (MPDSR) to enable a country’s use of audit data to track and prevent maternal perinatal deaths [15,16]. Accurate information about causes of death is needed to plan interventions to end preventable deaths. WHO released different guidelines [15,16,17] which provide guidance to countries for establishing and implementing a system of identifying and analyzing maternal and perinatal deaths, and implementing recommendations to eliminate preventable deaths. 

Few countries have robust operational MPDSR systems, particularly for maternal death notification. In some countries, MPDSR systems have been designed and/or are being implemented as standalone activities rather than as one among many important elements of goal-oriented quality improvement efforts focused on improving coverage, quality, equity, and access to care to reduce preventable maternal and perinatal morbidity and mortality [8,18,19,20]. 

With all eyes focused on achieving the Sustainable Development Goals (SDGs), Rwanda is looking to accelerate efforts to improve outcomes for women and neonates. Rwanda aims to further reduce its maternal mortality ratio from 210 per 100,000 live births in 2014/15 to 126, and infant mortality rate from 32 per 1000 live births in 2014/15 to 22.5 by 2024 [21]. We aimed to assess experiences in implementing maternal and perinatal death review, and/or integrated MPDSR processes in Rwanda by identifying factors that have affected its implementation. 

## 2. Materials and Methods 

Rwanda is the most densely populated country in Africa with a population of 12 million inhabitants and skilled birth attendance of 91% [11]. Its health system is decentralized and includes community health workers, health posts, health centers, district hospitals, provincial hospitals, and referral hospitals. The main medical services offered are paid for using community-based health insurance (commonly known as mutuelles de santé). 

The assessment team purposively sampled health facilities that had experience in conducting maternal and/or perinatal death reviews and/or implementing formal MPDSR processes or policies. A total of 13 health facilities (including 10 hospitals and three health centers) from 11 of Rwanda’s 30 districts participated in the assessment. All five provinces in the country were represented. Data were collected from December 2016 to January 2017. 

We applied mixed methods following the six-step audit cycle for MPDSR [17] as detailed in Figure S1. Qualitative methods included desk reviews of existing strategic documents such as maternal death surveillance and response (MDSR) guidelines, the health sector strategic plan, the neonatal protocol, the 2015 Rwanda demographic and health survey (DHS), the 2015 Rwanda health sector policy, and the Rwanda public health facility service package. In addition, the assessment included 13 facility observations and 23 key informant interviews (KIIs). Data were collected by maternal health experts and trained data collectors using a standard tool adapted from a study of Kangaroo Mother Care Implementation progress developed and tested by the South African Medical Research Council’s Unit for Maternal and Infant Health Care Strategies. The tool was translated into Kinyarwanda prior to the interviews. Facility observations involved checking available documentation to ensure existence of facility MPDSR committees, meeting schedules as well as their meeting notes. Based on respondents’ language preferences, interviews were conducted in English and/or Kinyarwanda. Interview notes were taken in English. 

Quantitative methods included scoring of responses from semi-structured in-person interviews composed of 64 questions as well as document review with facility-based staff involved in the mortality audit process. The questions were directed to health providers and facility managers involved in death audits. The questions focused on the following: when death audits started at their facilities, whether death audits were integrated and guidelines available, coordination of maternal and perinatal death audits, identification of deaths, collection and analysis of information about maternal and perinatal deaths, making recommendations, and implementing action plans for improvement (see Supplementary Materials File S3 for questionnaire). 

**Figure 1:**
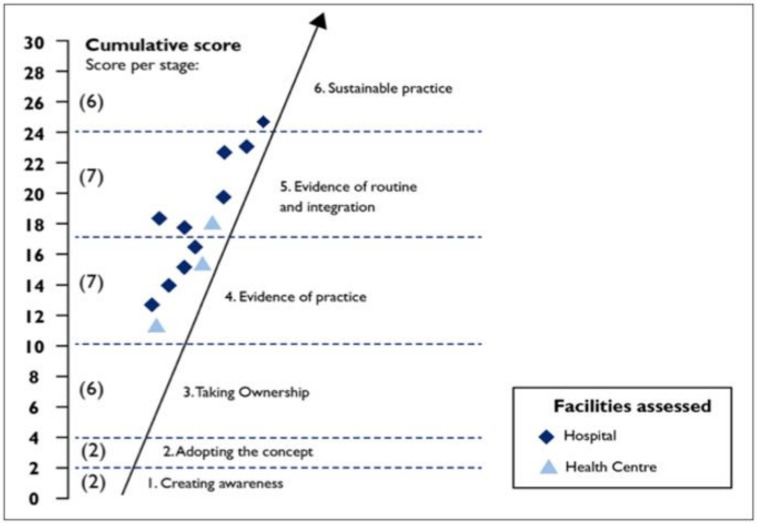
Facility score and stage of implementation.

A scoring scale to demonstrate the level of implementation of MPDSR at facility level was adapted from a study of Kangaroo Mother Care Implementation progress developed and tested by the South African Medical Research Council’s Unit for Maternal and Infant Health Care Strategies [22,23] as shown in Figure S2. Results were interpreted by means of a model with six stages of change, as shown in Figure 1, and facilities were scored out of 30. A facility score of less than 10 demonstrated that a facility was in the pre-implementation phase; a score greater than 10 and less than 17 demonstrated some level of implementation of MPDSR or evidence of MPDSR practice; a score above 17 and less than 24 demonstrated institutionalization of MPDSR through evidence of routine practice and integration; and scores higher than 24 showed sustainable MPDSR practice. 

The Rwanda National Ethics Committee approved the study protocol and tools (No. 874/RNEC/2016). The study was also determined to be non-human subjects research by the Johns Hopkins Bloomberg School of Public Health institutional review board. The data collected in this assessment did not include any personal identifiers from respondents. The questions in the tools gathered data on the current state of practice and did not require respondents to provide personal reflection or opinions, nor did assessors anticipate any risks associated with participation. Forms, registers, and meeting minutes collected did not include any identifying information of cases discussed through the MPDSR process. Before conducting KIIs, interviewers obtained oral consent by reading an oral consent script and asking the participant for a response. 

## 3. Results

### 3.1. Implementation Progress Scores

Overall, the MPDSR implementation status among the 13 facilities assessed scored between 11.75 and 24.21 (out of 30). The mean facility score was 17.30. All the facilities were in stage four or higher in terms of implementation of MPDSR. At the time of the assessment, six facilities demonstrated evidence of practice (stage four), six demonstrated evidence of routine practice and integration (stage five), while one facility demonstrated sustainable practice (stage six; Figure 1). 

### 3.2. The 6-Step Audit Cycle of MPDSR 

#### 3.2.1. Step 1: Identify Deaths 

The majority of the facilities reported a formal system for reviewing maternal deaths using documents from labor and delivery (*n* = 12/13; 92%), yet when asked specifically about stillbirths, three of those facilities did not provide a response and two facilities reported no formal system. Sources for identifying facility deaths across the 13 facilities are presented in Table 1, in which it is evident that facilities do not use source documents from postnatal care, outpatient, neonatology, antenatal care, etc. to identify maternal and perinatal deaths. 

#### 3.2.2. Step 2: Collect Information 

Five out of 13 facilities reported that medical records and registers were incomplete, making it difficult to assess cause of death and contributing factors. Seven facilities (54%) were involved more generally in efforts to improve the organization of medical records and registers, as recommended by the Ministry of Health (MOH). Efforts included using an electronic recording system, training staff on clinical documentation, and developing policies and procedures for patient assessments. 

#### 3.2.3. Step 3: Analyze Results 

Facility respondents reported that every MPDSR review meeting started with a review of the previous meeting’s recommendations. All facilities reported reviewing every maternal death during the meeting. The process of reviewing a new case involved reading aloud the questions on the audit form and looking through the patient chart to find the information needed to complete the audit form. The committee invited the attending doctor or nurse at the time of death for the audit process. 

After completing the background data and obstetric characteristics, the committee discussed the cause of death and whether the death was preventable. Assessors observed that some notification forms were not completed accurately, with some preventable deaths classified as unpreventable, signifying insufficient capacity of health workers to complete the forms. In addition, committees did not complete action plans for preventable cases as they should have done. The facility MPDSR committees did not use the International Classification of Disease-10 (ICD-10) to code deaths, and interviewed staff demonstrated limited knowledge of what the ICD-10 is and how to use it. Facility informants reported using the checklist system provided on the reporting form, which includes a space for distinguishing between direct and indirect obstetric causes of death. The chairperson of each MPDSR committee at all levels of the review process verifies completeness and accuracy of the maternal death report and requests additional information, if necessary. 

Nine out of 13 facilities indicated that the review process could result in a change to the cause of death as compared to the cause of death recorded in the facility records. Facilities described different processes for reconciling any discrepancies: one facility reported that the head nurse makes a change in the register and the data manager changes the report; another stated that they contact the MOH by phone after making a change to the register. During one of the interviews, a respondent indicated that effective communication was essential to ensure identification of maternal deaths.

“Effective communication is essential for ensuring accountability and the complete identification of maternal deaths. Now health care providers are more accountable and more prepared to deal with emergency and to work as a team to save the child and mother’s lives”.—Key informant 17.

Only three of the facilities reported sharing death data and trends during the review meetings. Nine of the 13 facilities reported that they took minutes of the meetings, and five were able to show documentation upon request. Some respondents also reported that not all members are always available for the audit meetings.

“Not all the time the member of the death auditing committee will be available for an auditing meeting due to daily workload”.—Key informant 11.

#### 3.2.4. Step 4: Recommend Solutions

In ten out of the 13 facilities assessed, action plans to address modifiable factors were developed by the MPDSR committee. Facility staff could provide examples of these action plans upon request; however, the action plans varied in level of completeness and detail. Sampled staff members could provide examples of linking modifiable factors to solutions. For example, one facility reported that, in most cases, the women referred from health centers to hospitals were already in critical condition. As a result, the facility decided to pair less-experienced providers with more-experienced providers so that the less-experienced ones could build up their skills to manage such cases in the future. Another example was where a woman died at the hospital because of complications/tears that the health center could not manage. In response, the hospital decided to assign midwives to accompany mothers in ambulances while traveling to the referring health facilities. Another facility reported an occasion when four women arrived at a labor and delivery ward where there were only three nurses and midwives working the night shift. The birth outcomes of the deliveries included one case of birth asphyxia, a death, and a case of fetal distress. Based on the audit review, the facility added more staff to the night shift and staff were required to complete rounds before each shift. A respondent at another facility noted that the number of neonatal deaths was decreasing due to the death auditing process. Citing the figures available at the health facility before implementation of death audits, the respondent stated that the facility used to record 13 to 15 neonatal deaths per month, but records show improvements of 8 to 10 neonatal deaths per month after implementing MPDSR. 

#### 3.2.5. Step 5: Implement Recommendations 

In five hospitals, recommendations were prioritized based on those which can be achieved at that particular facility as well as others that seemed rapid and feasible to achieve. One hospital, which indicated that it did not prioritize recommendations, reported that the medical director wrote a letter to the MOH requesting support for implementing its recommendations. 

In nine facilities, an individual was assigned to follow up on recommendations from the audit committee. Three facilities reported that the chairperson of the MPDSR committee assigned the person responsible for follow-up, based on expertise or specialty. Ten facilities were unable to describe a formal process for reporting back to the committee on the status of recommendations apart from reviewing minutes at the next mortality audit meeting. Lack of community engagement and heavy workload resulting in low motivation and other factors, as shown in Table 2, emerged as barriers to implementing MPDSR. Thus, to improve MPDSR processes, facilities suggested the following changes: creating linkages with neighboring communities, providing motivation and incentives for staff involved in MPDSR, hiring and retention of qualified staff, and providing additional training for staff. 

Eight facilities reported that they linked MPDSR to quality improvement (QI) activities within their facilities. One facility noted that the MPDSR committee attempted to work with the QI committee on quality-related recommendations. Another stated that most of the audit committee’s recommendations involve the QI team in the implementation process. 

Although eight facilities noted they communicated success stories, none had a process for formally documenting and reporting success stories. Five facilities reported sharing success stories during staff meetings but not through a formal or uniform system. In terms of feeding back recommendations from the facility-based death reviews to the community, only four of the facilities assessed reported doing this. Instead, recommendations to the community were typically shared with community health workers (CHWs) to disseminate to the community. One respondent mentioned that:

“One of the most challenging parts of the review process is the formulation of appropriate recommendations, but this step is critical to successful MPDSR”.—Key informant 5.

#### 3.2.6. Step 6: Evaluate and Refine 

The process of reporting back to the review committee on the status of recommendations and implementation was not the same across the visited facilities. At all facilities, clinical meetings included discussion of recommendations related to maternal and perinatal death audits, while monthly coordination meetings included the recommendations related to the health center and community. Despite verbal reports that recommendations were being implemented, only two facilities showed written feedback from the audit committees. The effectiveness of the death auditing committee and implementation of the recommendations depends on close follow-up of the clinical director who oversees the program. One of the respondents expressed that this is a key step in the process.

“Providing information about preventable factors that contribute to maternal death and using information to guide actions is key for preventing similar death in the future”—Key informant 19.

## 4. Discussion

This assessment aimed to assess and document the implementation and practice of MPDSR in Rwanda. Rwanda began implementing maternal death audits (MDAs) before perinatal death audits, however, the assessment found that MPDSR committees at the hospital and health center level are now routinely conducting both types of audits. Audit committees are also interdisciplinary in nature and the observed level of implementation of maternal and perinatal death audits was impressive. Rwanda is among the countries reported by WHO to have a national policy for notification and review of maternal deaths [24]. Rwanda has a very strong political will to adapt and implement high impact interventions to improve maternal and child health services. Strong political will was identified as a precursor of development and implementation of various policies and interventions aimed to improve maternal and child health and strengthen national health systems [18,19,25]. On the contrary, for many countries, lack of political buy-in and long-term vision has been identified as a challenge and barrier in implementation of MPDSR [8,20,24]. 

Although we found a strong political will to improve maternal and newborn health, we also identified challenges. This assessment revealed the need to strengthen several aspects of MPDSR implementation in Rwanda. The minimum aim of the MPDSR system is to identify all births, maternal deaths, stillbirths, and neonatal deaths that occur, whether in the labor ward, in other departments within a health facility, or in the community. Ninety-two percent and 100% of facilities reported a formal system of reviewing maternal deaths and perinatal deaths, respectively, indicating evidence of practice and integration of the death audits with their other routine work. Nonetheless, this assessment revealed that there was no formal system for perinatal mortality audits in Rwanda. A lot of emphasis was previously put on reducing maternal mortality; and conducting maternal death audits was seen as a key contributing factor. Health facilities did not have standard guidance related to perinatal death audits. Therefore, the fact that implementation of MPDSR started much earlier than perinatal mortality audits may explain why the latter was not well implemented by the time of the assessment. Findings from other settings suggested that for many cases of perinatal mortality the cause of death is never established [17,26,27], suggesting that perinatal death audits are probably not done. Also, a few high-income countries have an active national program of stillbirth audits [28]. Although facility-based deaths were easily identified, the findings showed that only a third of the health centers assessed and tracked deaths within the community, and yet the information was identified as key in avoiding preventable deaths [29,30,31]. 

Accuracy of data on death notification forms is key to successful implementation of mortality reviews. With more than a third of facilities reporting that patient charts and records were often incomplete, there is need for revision so that providers are not burdened with entering unnecessary information. Furthermore, facility staff may need training in completing patient charts and records. Accurate charts will ensure that audit committees have the information they need to analyze and determine the cause of death as well as contributing factors. Similarly, audit committees should be encouraged to display and analyze data and trends during their audit meetings to understand better the quality of service delivery at their facilities, identify common causes of deaths, and help prioritize responses [32]. 

Findings showed that most facilities identified maternal deaths using labor and delivery registers yet maternal deaths could happen in other services. This could lead to under estimation of the actual maternal deaths due to inefficient or incomplete systems of notification highlighting challenges and barriers to the implementation of MPDSR [24]. Assessors noted that committees did not always classify causes of deaths accurately on reporting forms. As such, the MOH and district level managers should consider providing additional training on cause-of-death classification, aligned with the ICD-MM (maternal mortality) and ICD-PM (perinatal mortality), because accurate classification informs the type and quality of responses developed by audit committees. The MOH should provide guidance for purposeful selection of perinatal deaths to ensure that facility staff review representative cases. As they do for maternal deaths, national guidelines should also provide a timeline for reporting perinatal deaths. 

Audit committees made recommendations based on identified causes and followed through the response function (implementation of the action plan) which is a key component of implementing MPDSR. Though facilities were able to give examples of changes resulting from MPDSR, action plans reviewed during the assessment varied in quality. Furthermore, there were no systematic processes for tracking implementation of action plans. The response and monitoring of recommendations made during audit sessions is still an issue in countries where maternal death review systems are not well established [25,27]. In some countries with well-established national systems, reports with recommendations are systematically disseminated to relevant entities for implementation with a clear process of monitoring implementation at different levels [18,26,27]. However, timely production of those reports may not work for Rwanda due to the high number of maternal deaths that still occur each year. 

Documenting success stories from audits and disseminating them within facilities and communities may be a motivational tool for audit committee members. However, this is not being implemented by all the facilities assessed. Facility respondents reported significant barriers to the implementation of recommendations, including lack of motivation and heavy workloads. Supporting committees to identify and prioritize easily achievable recommendations may motivate committee members, as could recognizing staff efforts. These challenges are common in many similar settings in Rwanda. In addition to challenges found in our assessment, another assessment in Uganda [33] noted that health providers felt that MPDSR committee members lacked adequate support in terms of supervision and financial motivation. Challenges to MPDSR included: heavy workload to health workers, high number of perinatal deaths, and non-implementation of recommendations [3,15,17]. 

One of the goals of MPDSR is to improve the quality of care provided to mothers and newborns. The assessment found that QI committees, tasked with developing strategies to improve the quality of health care, exist within many health facilities but work independently of MPDSR committees. Facilities should integrate the work of QI and MPDSR committees to help facilitate implementation of recommendations. 

The national guideline states that the goal of the audit meetings is not to blame individuals, but instead to facilitate learning and avoid preventable deaths. However, this assessment shows that more could be done to promote the no name, no blame principle. Among barriers and challenges to implement MPDSR identified in the global implementation of Maternal Death Surveillance and Response report [24], a blame culture in some places inhibits health professionals and others from participating fully in the MPDSR process [3,34,35]. Adopting clear codes of conduct differentiating between the mortality audit and disciplinary processes and emphasizing confidentiality may create a better atmosphere for staff to share information without fear of blame or punishment [25,30]. 

Regarding limitations, the assessment was focused on a limited number of health facilities, and interviews focused mainly on the process of conducting mortality audit at the facility level. Most of the information collected was based on self-report by the informants present at each health facility on the day of data collection, therefore the feedback depended on who was available to interview on the day of the visit. Although considered socially correct, some of the views expressed may not necessarily reflect those of other health care staff. 

## 5. Conclusions

Rwanda has a strong political will to reduce maternal and newborn deaths as evidenced by the level of implementation of MPDSR among other efforts in the country. The assessment showed evidence of practice and of sustainability of MPDSR, although there was variability in level of implementation from facility to facility. The assessment did not find evidence for a formal system for perinatal death audits, although maternal death audits are conducted regularly, indicating the need to develop and disseminate perinatal death audit guidelines and integrate them with maternal death audits. Health providers also lacked enough knowledge to identify and properly classify the cause of death, indicating a gap in capacity. There is need to hire, train, retain, and motivate staff involved in death audits to ensure proper implementation of MPDSR as a quality improvement approach, and this calls for integration of MPDSR with QI activities at facility level as well as linkages with communities for feedback. Facilities should be encouraged to properly document cases and share best practices to foster learning and skill building to prevent avoidable maternal and newborn deaths in the future. Well implemented MPDSR systems will contribute to Rwanda’s efforts to reduce preventable maternal and perinatal deaths as well as achieve SDG 3. 

## Figures and Tables

**Table 1:** Sources for identifying health facility deaths.

Register Source	Number (Percentage) of Health Facilities (HFs)
	13 (%)
Labor and delivery	12 (92)
Postnatal	7 (54)
Adult inpatient	5 (38)
Neonatal	5 (38)
Ambulatory and emergency	4 (31)
Antenatal care	2 (15)
Outpatient	1 (8)

**Table 2:** Barriers to implementing maternal and perinatal deaths surveillance and response (MPDSR).

Barriers	Number (Percentage) of HFs
	13 (%)
Lack of community engagement	7 (54)
Inadequate personnel with necessary up-to-date clinical competencies	5 (38)
Limited number of qualified personnel	4 (31)
Inadequate support from facility leadership	3 (23)
Inadequate support from district leadership	3 (23)
Lack of communication across levels	3 (23)
Inadequate referral system	2 (15)
Limited resources/finances	2 (15)
Lack of essential commodities	1 (8)
Existence of harmful local practices	1 (8)

## Supplementary Materials

The following are available online at https://www.mdpi.com/1660-4601/17/12/4376/s1; Figure S1: The six-step audit cycle for maternal and perinatal death surveillance and response, Figure S2: Implementation progress schematic scoring scale adopted from South African Medical Research Council [22,23], File S3: Questionnaire.
