# Supplementary material for: Assessing Implementation of Maternal and Perinatal Death Surveillance and Response in Rwanda
Source: Int J Environ Res Public Health. 2020 Jun 18;17(12):4376. doi: 10.3390/ijerph17124376 (PMC7345772; doi:10.3390/ijerph17124376)
Supplement: Supplementary file 1 [file ijerph-17-04376-s001.pdf]

Supplementary materials

Supplementary information

Figure S1: The six-step audit cycle for maternal and perinatal death surveillance and response

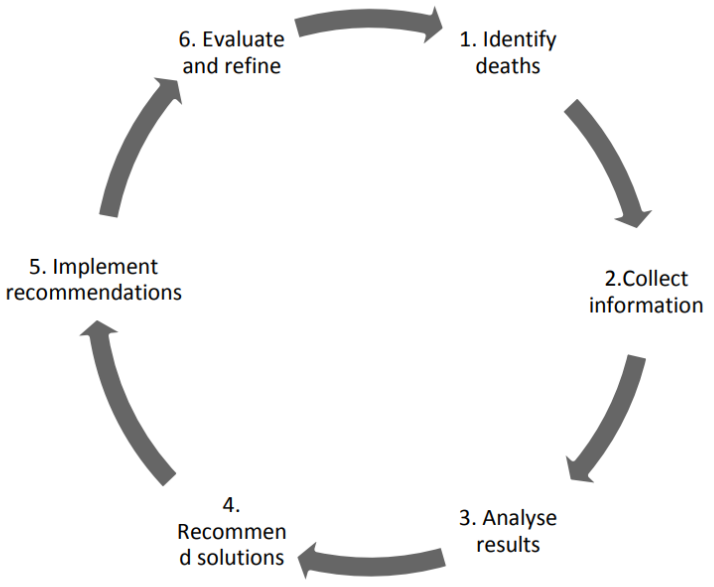

Figure S2. Implementation progress schematic scoring scale adopted from South African Medical Research Council [19, 20].

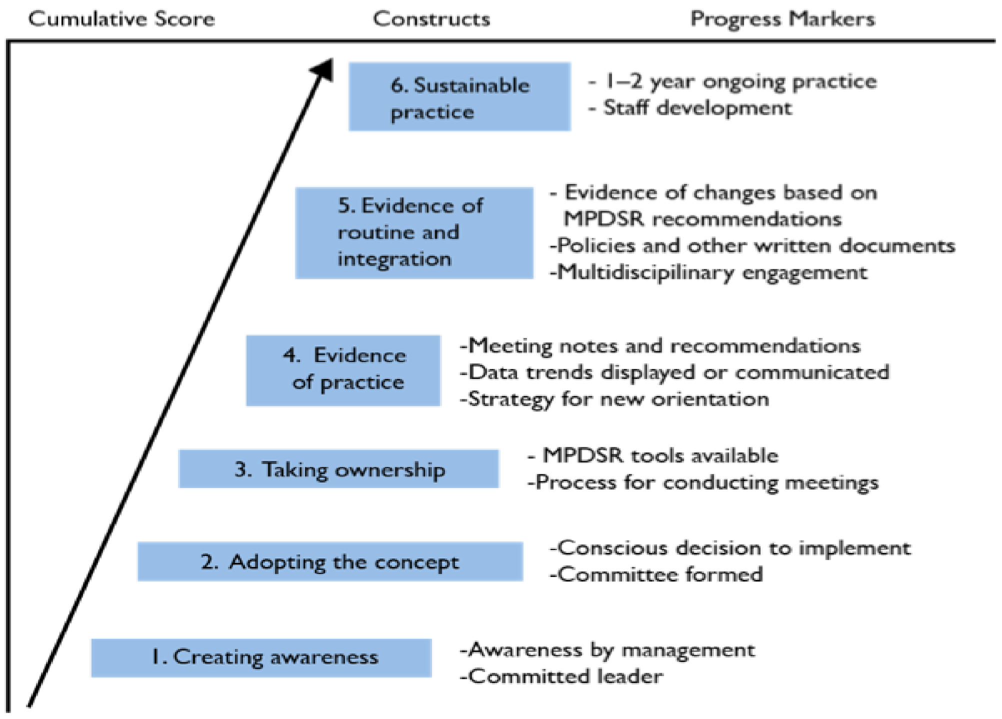

S3- Questionnaire: guiding questions

Adapted from MRCSA KMC progress monitoring tool, version 5

Maternal and Perinatal Death Surveillance and Response (MPDSR) Implementation Progress Monitoring Tool (Version 1)

Guidelines for monitors / assessors:

- Please use separate forms for each individual respondent.
- Unless the maternal and perinatal review committees are combined into one process led by the same individual, please use separate forms to capture information relating to the maternal death review process and the perinatal death review process.
- Note that each facility might have a different name for the audit / review / surveillance and response team. Try to use local terminology as much as possible.
- Be sure to probe about what deaths are captured, especially in relation to stillbirth, perinatal, and child deaths as these processes are likely to be less well known than the systems for maternal deaths.
- Request to make photocopies of all written documents related to M/PDSR, especially where noted in the questionnaire below. If photocopies are not available, ask for permission to photograph the documents for record purposes.
- Ask for photocopies of samples of data collection forms, meeting minutes, action items, and relevant material. If copies are not available, ask for permission to photograph the documents for record purposes. Be sensitive to ethical issues and patient privacy. If you need to photograph a document with identifying details, cover the names or details with a piece of paper before taking the picture in order to preserve confidentiality.
- Ask for permission from the hospital or nursing services manager to take pictures of the hospital, staff or records. (Pictures of staff members are only to be taken if they also give their verbal consent.)
- Mark each of the documents you take away with a date and the name of the hospital, where applicable.
- Each monitor/assessor fills in his/her own checklist and the results are compared and consolidated afterwards on one checklist, which is then marked as “FINAL”.

Instructions:

- Tick or cross only applicable boxes.
- Complete the “comments” and “observations” sections if something important or striking is mentioned or observed that may be informative to understanding a particular phenomenon. Use the back of the questionnaire form if necessary.
- Where possible, complete “specify”, “describe”, “explain” and “elaborate” where the associated response is ticked.

Name of progress monitor / assessor: ……………………………………………   Date: ……………………………

OBSERVATIONS AND QUESTIONS TO ASK HEALTH WORKER INFORMANTS

A. **HEALTH CARE FACILITY**

1. District: ………………………………………………

2. Name of facility: ………………………………………………………..

3. Level of facility (teaching/ referral/ provincial/ district / health centre):
   ………………………………………………………….

4. Is there a MDSR coordinator or stakeholder at the facility?
☐ Yes ☐ No ☐ Unsure

   a. Job title: …………………………………… *(write none if MDSR is not done at this facility)*

5. Is there a PDSR coordinator or stakeholder at the facility?
☐ Yes ☐ No ☐ Same as MDSR coordinator ☐ Unsure

   a. Job title: …………………………………… *(write none if PDSR is not done at this facility)*

6. Does the coordinator(s) have other responsibilities (e.g. information officer, QI focal point, nurse, etc.):
   If yes, please specify the other responsibilities) ☐ Yes ☐ No

……

7. Does the facility have a formal system for reviewing maternal deaths, stillbirths, and/or neonatal deaths?
   Maternal deaths: ☐ Yes ☐ No ☐ Unsure
   Perinatal deaths: ☐ Yes ☐ No ☐ Unsure
   Stillbirths: ☐ Yes ☐ No ☐ Unsure
   Neonatal deaths: ☐ Yes ☐ No ☐ Unsure

   Comments: ………

   ……

   Near-misses? ☐ Yes ☐ No ☐ Unsure

   Comments: ………

   ……

8. Does the facility have a steering committee for MPDSR? ☐ Yes ☐ No ☐ Unsure
   If yes, please describe (e.g. maternal, perinatal, both, separate, etc):

   ……

   ……

   ……

B. **HISTORY OF MPDSR IMPLEMENTATION**

9. When was MDSR started at the facility? ……………………………………………………………………………………………

10. When was PDSR started at the facility? ……………………………………………………………………………………………

11. We would like to know more about the process that was followed. Where did the decision to undertake M/PDSR originate? (e.g. district, facility, or national level)

………

12. Was there a specific occasion or meeting where the decision to implement MPDSR was taken?

☐ Yes ☐ No ☐ Unsure If yes, approximate date:
……………………………………………………………………

13. Was there an implementation or action plan established?

☐ Yes ☐ No ☐ Unsure

14. Is there written minutes or documentation of the decision?

☐ Yes ☐ No ☐ Unsure *(If Yes, ask if it would be possible to see a copy. Ensure that all personally identifiable information is removed or obscured)*

Documentation seen ☐ Yes ☐ No
Document received / photographed ☐ Yes ☐ No

15. *If M/PDSR is not implemented yet:* has a formal decision for M/PDSR implementation been made yet?
☐ Yes ☐ No ☐ Unsure

If yes, describe: ………

16. Before starting MPDSR, did the facility systematically document the following baseline data?

Number of maternal deaths: ☐ Yes ☐ No ☐ Unsure

Cause of maternal deaths: ☐ Yes ☐ No ☐ Unsure

Number of perinatal deaths: ☐ Yes ☐ No ☐ Unsure

Cause of perinatal deaths: ☐ Yes ☐ No ☐ Unsure

C. MPDSR ROLE-PLAYERS

17. Has anyone in facility or district leadership signed a commitment or undertaken an agreement that s/he would ensure that M/PDSR is implemented in the facility?

☐ Yes ☐ No ☐ Unsure If yes, specify title: …………………………………………………………………

18. What kind of support did you get from the following people? *(specify type of support, or write none, or not applicable if the post does not exist at the facility or district)*

District Director of Health/Health Officer: …………………………………………………………………

District M&E Officer: …………………………………………………………………

Facility Director or In-Charge: …………………………………………………………………

Matron / Nursing service manager: …………………………………………………………………

Unit manager (neonatal unit or maternity): …………………………………………………………………

Obstetrics: …………………………………………………………………

Paediatrics: …………………………………………………………………

Data Manager: …………………………………………………………………

Head of Quality Management Committee …………………………………………………………………

Other, specify: …………………………………………… …………………………………………………………………

19. Do you have educational activities in your facility to introduce MPDSR to staff members?

☐ Yes ☐ No ☐ Unsure If yes, describe: ……………………………………………………………………………..

Are activities internal, or led by district or national? …………………………………………………………………..

Are activities held on-site or off-site? ……………………………………………………………………………………..

20. Approximately how many staff members are currently involved in MPDSR? …......................................

Managers (e.g. facility administrators) ………..

Clinicians (doctors or medical officers) ………..

Nurses/midwives ………..

Other (specify) …………………………………….. ………..

21. Have you received support (financial or in-kind) from the hospital or district budget to establish MPDSR?

☐ Yes ☐ No ☐ Unsure If yes, describe: ……………………………………………………………………………..

D. MPDSR PRACTICE

22. Are there any *written* policies, guidelines or protocols regarding the practice of MPDSR?

☐ Yes ☐ No ☐ Unsure If yes, describe: ………………………………………………………………………….
(Note whether the document is specific to the facility, district or national level. Obtain a copy or take a photo if possible)

MPDSR CYCLE: IDENTIFYING DEATHS

23. How are deaths identified? (*Let the respondent answer first, then probe for different areas of facility, especially for maternal deaths as these are more likely to occur in different areas of the facility)*
    - ☐ ANC register
    - ☐ Ambulatory emergency care area
    - ☐ General adult inpatient ward
    - ☐ Labour and delivery register
    - ☐ Outpatient department register
    - ☐ Postnatal register
    - ☐ Neonatal register
    - ☐ Other, specify: …….

24. Are maternal and/or perinatal deaths that occur in the community documented at this facility?
☐ Yes ☐ No ☐ Unsure If yes, what is the process for learning about and documenting these?

……

NB: expand understanding about current community data collection and follow-up

MPDSR CYCLE: COLLECTING INFORMATION

25. How is information about maternal and/or perinatal deaths collected and summarised for MPDSR?

……

……

Ask to see a copy of the forms used (obtain a copy or request to take a photograph, specifically capturing the sections where cause of death, modifiable factors, and solutions are recorded)

26. What documents are used to compile cases for mortality audit meetings?
    - ☐ Patient charts / case notes
    - ☐ Registers
    - ☐ None
    - ☐ Other, specify: …….

27. In your opinion, do the medical records and registers capture the necessary information for assessment of cause of death and contributing factors for maternal and perinatal deaths?

……

……

28. Is your facility involved in any efforts to improve the organization of medical records and registers (e.g. standardization of records with minimum essential data points)?

……

……

29. What system is used to classify cause of death on the mortality audit forms?
    - ☐ ICD-10
    - ☐ Modified ICD-10
    - ☐ None
    - ☐ Other, specify: ………..

30. What system is used to classify modifiable factors or sub-standard care?
    - ☐ 3 delays
    - ☐ Root cause analysis
    - ☐ Patient – Provider – Administrator
    - ☐ None
    - ☐ Other, specify: ………..

31. Are there any statistics related to MPDSR displayed somewhere (e.g. on a wall)?

☐ Yes ☐ No ☐ Unsure If yes, describe what indicators are included:

……

32. Are there official channels through which MPDSR findings are reported to different levels of management on a regular basis?

☐ Yes ☐ No ☐ Unsure If yes, where are the findings sent?

……
(Obtain a copy or request to take a photograph of the reporting template from the health facility to other levels within the system)

MPDSR CYCLE: ANALYSING DATA AND PRESENTING RESULTS

33. How frequently do mortality audit meetings take place?………………………………………………………………………..

34. Who (positions/job titles) are invited to attend?………………………………………………………………………………………..

35. Is attendance mandatory? ☐ Yes ☐ No ☐ Unsure

36. What is the title of the most senior staff member or administrator normally present?…………………………

37. What is the title of the staff or administrator who runs the meetings?…………………………………………………

38. What is presented at the meetings (describe what happens at the meetings)?

……

……

39. Is every death reviewed or is a sample of deaths selected for discussion? ……………………………………………..

40. If a sample of deaths of deaths is selected what criteria are used to decide which deaths get reviewed?

……….

41. What trend data or statistics are routinely presented, if any? ……………………………………………………………..

42. Are meeting minutes taken? ☐ Yes ☐ No ☐ Unsure
(If yes, obtain a copy or request to take a photograph of recent meeting minutes. Ensure that all personally identifiable information is removed or obscured)

MPDSR CYLCE: RECOMMENDING SOLUTIONS

43. How are modifiable factors linked to solutions in your MPDSR process?

……

……

44. How does the mortality review team identify and prioritize recommendations?

……

……

45. Is an action plan developed as part of the review process?

☐ Yes ☐ No ☐ Unsure If yes, describe what the action plan entails:

……

MPDSR CYCLE: IMPLEMENTING CHANGES

46. Does the mortality review process ever result in a change to the cause of death as compared to the cause of death recorded in the facility records (e.g. vital statistics report, maternity register, maternity monthly report, etc.)?

☐ Yes ☐ No ☐ Unsure If yes, how is this reconciled?

……

47. Are individuals assigned to follow up on specific recommendations?

☐ Yes ☐ No ☐ Unsure If yes, how is this assigned?

……

48. What is the process for reporting back to the review team on the status of recommendations?

……

49. Is there a written documentation system for tracking the follow-up on specific recommendations?

☐ Yes ☐ No ☐ Unsure (*If yes, obtain a copy or request to take a photograph*)

50. In your opinion, what are some barriers to ensuring recommendations are implemented following mortality review (e.g. completing the "Response" portion of MPDSR)?
    - ☐ MOH leadership/support
    - ☐ Facility leadership/support
    - ☐ District leadership/support
    - ☐ Lack of communication across levels
    - ☐ Inadequate referral system
    - ☐ Availability of essential commodities
    - ☐ Availability of qualified personnel
    - ☐ Availability of personnel with necessary up to date clinical competencies
    - ☐ Availability of resources/finances
    - ☐ Lack of community engagement
    - ☐ Harmful local practices
    - ☐ Other (describe) ……

51. Do you regularly link MPDSR to any other quality improvement activities in your facility?

……

52. Are success stories communicated? ☐ Yes ☐ No ☐ Unsure

If yes, how: ……

53. Are the recommendations from facility-based death reviews fed back to the community in any way?

……

AVOIDING BLAME AND ENSURING CONFIDENTIALITY

54. How do you ensure staff protection during the mortality review process?

……

55. Are the names of individual staff members included in audit reports?

☐ Yes ☐ No ☐ Unsure If yes, please describe:

……

56. Is there any connection to professional disciplinary action and the MPDSR system?

☐ Yes ☐ No ☐ Unsure If yes, please describe:

……

57. Do you see any risks associated with the M/PDSR process?

☐ Yes ☐ No ☐ Unsure If yes, please describe:

………

E. CASE STUDY QUESTIONS

58. What do you think is working well in your facility regarding MPDSR? What were the main factors that facilitated implementation of MPDSR in your facility?

………

………

59. What are / were some of the barriers / obstacles to the implementation of MPDSR?

………

………

60. What changes would be most helpful to improve the utility of MPDSR in your facility?

………

………

61. Can you tell us about a time where the recommendations made during the mortality audit process resulted in a change in how care was provided?

………

………

………

62. Approximately how much time (hours) does the MPDSR committee spend per month on all activities related to MPDSR in your facility?

………

63. Sometimes mortality audit can be a demoralising activity for staff. How do you maintain morale in meetings?

………

………

64. In your view how useful is MPDSR for improving the quality of care and health outcomes for women and newborns in your facility?

………

………

ASSESSOR'S GENERAL OBSERVATIONS AND IMPRESSIONS

Impressions regarding respondent's recall of the history of implementation:
☐ Good recall *☐ Some recall* *☐ No recall*

Comments:
……

Impressions regarding the intensity of involvement of facility senior management in conducting MPDSR

☐ A lot of involvement and/or support (moral, material, etc)

☐ Some involvement and/or support (moral, material, etc)

☐ Neutrality / Little support

☐ Resistance

Comments:
……

Impressions of the quality of data captured in MPDSR summary forms

☐ Excellent

☐ Average

☐ Poor

Comments:
……

Impressions of the quality of recommendations contained in the review meeting notes

☐ Excellent

☐ Average

☐ Poor

Comments:
……

Impressions of the quality of follow up actions

☐ Excellent

☐ Average

☐ Poor

Comments:
……

Other comments and observations

……

………

COMMENTS FOR FACILITY (FOR IMMEDIATE FEEDBACK)

GENERAL IMPRESSIONS OF MONITOR/ASSESSOR

………

………

ASSESSOR'S RECOMMENDATIONS FOR FACILITY LEVEL CONSIDERATION

………

………

IDEAS FOR POLICY MAKERS AND OTHER LEVELS OF MANAGEMENT

………

………

NAME OF ASSESSOR SIGNATURE

DATE
